# Treatment of metastatic castration resistant prostate cancer with radium-223: a retrospective study at a US tertiary oncology center

**DOI:** 10.1038/s41391-020-00271-7

**Published:** 2020-08-19

**Authors:** Rana R. McKay, Rebecca Silver, Rachel H. Bhak, Caroline Korves, Mu Cheng, Sreevalsa Appukkuttan, Stacey J. Simmons, Mei Sheng Duh, Mary-Ellen Taplin

**Affiliations:** 1 Dana-Farber Cancer Institute, Boston, MA USA; 2 University of California San Diego, Moores Cancer Center, La Jolla, CA USA; 3 Analysis Group, Inc, Boston, MA USA; 4 Bayer Healthcare Pharmaceuticals, Whippany, NJ USA

**Keywords:** Cancer therapy, Prostate cancer

## Abstract

**Background:**

Guidelines for optimal sequencing of radium-223 and chemotherapy for metastatic castration resistant prostate cancer (mCRPC) do not exist. This study evaluated treatment patterns and overall survival (OS) among patients with mCRPC treated with radium-223 in an academic clinical setting.

**Methods:**

A retrospective study was conducted of bone metastases-predominant mCRPC patients treated with radium-223. Treatment patterns from 2013 to 2018 were evaluated in patients treated with radium-223 pre- vs. post-chemotherapy. OS was examined using Kaplan–Meier medians and 95% confidence intervals.

**Results:**

In total, 220 patients were treated with radium-223 (64 pre-chemotherapy, 83 post-chemotherapy, 73 no chemotherapy). Mean radium-223 injections per patient was 5.3 and 4.3 in the pre- vs. post-chemotherapy cohorts, respectively (*p* < 0.001). The number of chemotherapy cycles was similar for chemotherapy given pre- or post-radium-223. Mean line of mCRPC therapy of radium-223 was 3rd and 5th when given pre- and post-chemotherapy, respectively (*p* < 0.001). 41.8% patients were treated with radium-223 in combination with another mCRPC therapy, commonly abiraterone acetate (43.5%) or enzalutamide (52.2%). The majority received combination therapy for the duration of radium-223 treatment; 20.7% started another agent after radium-223 initiation; 20.7% initiated radium-223 while on established therapy. Median OS from first mCRPC treatment was 39.4 months (95% CI 33.0, 48.8) for patients with radium-223 pre-chemotherapy vs. 37.4 months (95% CI 32.0, 43.5) post-chemotherapy (and 35.2 months [95% CI 27.9, 43.3] vs. 32.0 months [95% CI 26.9, 36.0] for patients with radium-223 combination vs. monotherapy).

**Conclusions:**

This retrospective analysis of patients treated with radium-223 demonstrates that administration of radium-223 pre-chemotherapy increased likelihood of completion of radium-223 treatment. Radium-223 given pre- or post-chemotherapy and with or without combination therapy did not result in significant differences in OS. Additional studies are needed to determine the optimal sequencing strategy of mCRPC in the modern era.

## Introduction

The treatment landscape for metastatic castration resistant prostate cancer (mCRPC) is expanding and now includes chemotherapy, oral hormonal therapies, immunotherapy, and radiopharmaceutical therapy, which have demonstrated improvements in overall survival (OS) [1–8], as well as poly ADP-ribose polymerase (PARP) inhibitors which have demonstrated prostate-specific antigen (PSA) and soft tissue response among patients with homologous recombination repair gene alterations [9]. Radium-223 is an alpha emitting radiopharmaceutical approved in 2013 by the United States (US) Food and Drug Administration (FDA) for patients with CRPC with bone metastases [10]. Approval was based on the results of the ALSYMPCA trial [8] which demonstrated that radium-223 increased OS and delayed time to symptomatic skeletal events (SSEs); radium-223 was the first bone-targeting alpha therapy to show a survival advantage [11]. Given the predominance of bone metastases in patients with prostate cancer and the negative consequences of bone metastases on morbidity and mortality, targeting bone in men with prostate cancer improves outcomes for patients [8].

National Comprehensive Cancer Network guidelines list several treatments, including radium-223 and docetaxel, as category 1 therapies for first and subsequent treatment lines for mCRPC without visceral metastasis [12]. Despite the introduction of these therapies, level 1 data for optimal treatment sequencing of radium-223 and chemotherapy [1, 2, 13] are lacking. Currently, treatment sequencing decisions are made without treatment selection tools such as validated biomarkers of response [14] and are based on patient and provider discussions of patient goals and risk/benefit assessments. The aim of this study was to evaluate treatment patterns and outcomes among CRPC patients with bone metastasis treated with radium-223 in clinical practice. This study evaluates the contemporary use of radium-223 in the modern era amidst a growing landscape of treatment options for patients with metastatic CRPC. We hypothesized that patients receiving radium-223 pre-chemotherapy will have differential outcomes compared to patients receiving radium-223 post-chemotherapy. In addition, given potential for synergy, we hypothesized that patients receiving radium-223 in combination with other therapies compared to as monotherapy will have differences in outcomes.

## Methods

### Study design

This was a noninterventional, retrospective, longitudinal chart review study among patients with bone metastases-predominant mCRPC treated with radium-223 at a tertiary cancer center, Dana-Farber Cancer Institute (DFCI) in Boston, MA. Data from patient charts were reviewed from January to April 2019 and collected via a secure electronic data platform. Data were deidentified and complied with the patient confidentiality requirements of the Health Insurance Portability and Accountability Act. All study materials were approved by the Institutional Review Board at DFCI.

The index date was defined as the time of initiation of first mCRPC therapy, and the observation period spanned the index date until the date of last contact or death. Baseline demographic and clinical characteristics were captured prior to the index date. Outcomes were assessed during the observation period, including mCRPC treatment patterns (e.g., line of treatment for each therapy, number of radium-223 injections, number of cycles of chemotherapy, bone-targeting agents), PSA at initiation of each mCRPC treatment, reasons for treatment discontinuation, OS, and SSEs.

### Patient population

All patients who received at least one dose of radium-223 between 2013 and 2017 and did not have visceral metastases at the time of radium-223 were included in the study. Patients may have received pre-mCRPC treatments and mCRPC treatments as part of clinical trials or expanded access programs (EAP).

### Statistical analysis

Descriptive statistics were calculated using frequencies and proportions for categorical variables and means, standard deviations, and medians for continuous variables. For comparisons, *p* values were computed using a chi square test (or Fisher’s exact test as appropriate) for categorical variables and a Wilcoxon rank-sum test for continuous variables.

OS from time of initiation of first-line mCRPC treatment (and time from initiation of radium-223) until death from any cause were estimated using Kaplan–Meier analysis where patients who did not die were censored.

Analyses were conducted overall and for patients who received radium-223 pre- vs. post-chemotherapy for mCRPC. Data were also analyzed for patients treated with radium-223 as monotherapy and as combination therapy (radium-223 overlapping with other mCRPC therapies).

All analyses were performed using SAS 9.4 (SAS Institute, Cary, NC).

## Results

### Patient baseline and clinical characteristics

There were 220 patients treated with radium-223 who were included (Table 1). The median age at mCRPC diagnosis was 67.6 years. The majority of patients were Caucasian (*n* = 195, 88.6%). Cardiovascular disease was the most common comorbidity (*n* = 55, 25.0%). Among 220 patients treated with radium-223, 35 (15.9%) had an SSE prior to initiation of first-line mCRPC treatment. Mean ± SD PSA at initiation of first-line mCRPC treatment was 80.9 ± 220.5 ng/mL and at initiation of radium-223 was 271.5 ± 632.3 ng/mL. At initiation of radium-223, all 220 patients had bone metastasis, and 102 (46.4%) had lymph node metastasis.

**Table 1:** Patient demographic characteristics and clinical characteristics prior to initiation of radium-223.

	Patients treated with radium-223 (*N* = 220)	Patients treated with radium-223 and no chemotherapy (*N* = 73)	Patients treated with radium-223 pre-chemotherapy (*N* = 64)	Patients treated with radium-223 post-chemotherapy (*N* = 83)	*p* value*
Age at prostate cancer diagnosis (year), mean ± SD [median]	63.5 ± 9.3 [62.4]	68.5 ± 10.3 [68.7]	61.5 ± 8.0 [61.4]	60.7 ± 7.3 [60.1]	0.396
Prostate cancer diagnosis year, *n* (%)					
Prior to 2010	120 (54.5)	36 (49.3)	32 (50.0)	52 (62.7)	0.124
2010–2011	31 (14.1)	11 (15.1)	9 (14.1)	11 (13.3)	0.887
2012–2013	44 (20.0)	14 (19.2)	12 (18.8)	18 (21.7)	0.661
2014–2015	22 (10.0)	10 (13.7)	10 (15.6)	2 (2.4)	0.004*
2016–2017	3 (1.4)	2 (2.7)	1 (1.6)	0 (0.0)	0.435
Gleason score (sum of primary and secondary Gleason scores), *n* (%)					
≤6	19 (8.6)	6 (8.2)	5 (7.8)	8 (9.6)	0.699
7	55 (25.0)	17 (23.3)	18 (28.1)	20 (24.1)	0.580
≥8	112 (50.9)	29 (39.7)	37 (57.8)	46 (55.4)	0.772
Not available/Unknown	34 (15.5)	21 (28.8)	4 (6.3)	9 (10.8)	0.331
Age at mCRPC diagnosis (year), mean ± SD [median]	69.2 ± 8.6 [67.6]	74.8 ± 8.9 [75.4]	66.5 ± 7.8 [66.0]	66.2 ± 6.3 [65.2]	0.778
Missing, *n* (%)	1 (0.5)	0 (0.0)	1 (1.6)	0 (0)	
Metastatic disease diagnosis >30 days prior to CRPC, *n* (%)	132 (60.0)	46 (63.0)	40 (62.5)	46 (55.4)	0.388
Comorbidities at mCRPC diagnosis and 6 months prior, *n* (%)					
Cardiovascular disease	55 (25.0)	24 (32.9)	15 (23.4)	16 (19.3)	0.540
Diabetes	21 (9.5)	10 (13.7)	5 (7.8)	6 (7.2)	>0.999
Other cancers	21 (9.5)	13 (17.8)	4 (6.3)	4 (4.8)	0.728
Renal disease	13 (5.9)	7 (9.6)	1 (1.6)	5 (6.0)	0.233
Liver disease	3 (1.4)	2 (2.7)	1 (1.6)	0 (0.0)	0.435
No comorbidities	78 (44.6)	18 (31.0)	25 (49.0)	35 (53.0)	0.667
Not available/Unknown	7 (4.0)	0 (0.0)	2 (3.9)	5 (7.6)	0.467
Identification of metastasis at initiation of Radium-223, *n* (%)					
Bone	220 (100.0)	73 (100.0)	64 (100.0)	83 (100.0)	>0.999
Lymph node	102 (46.4)	29 (39.7)	37 (57.8)	36 (43.4)	0.083
Brain	1 (0.5)	0 (0.0)	0 (0.0)	1 (1.2)	>0.999
Race/Ethnicity, *n* (%)					
White	195 (88.6)	62 (84.9)	61 (95.3)	72 (86.7)	0.079
Black or African American	10 (4.5)	4 (5.5)	2 (3.1)	4 (4.8)	0.697
Hispanic or Latino	1 (0.5)	1 (1.4)	0 (0.0)	0 (0.0)	>0.999
Asian/Pacific Islander	2 (0.9)	0 (0.0)	0 (0.0)	2 (2.4)	0.505
Native American or American Indian	1 (0.5)	1 (1.4)	0 (0.0)	0 (0.0)	>0.999
Other	2 (0.9)	2 (2.7)	0 (0.0)	0 (0.0)	
Not available/Unknown	10 (4.5)	4 (5.5)	1 (1.6)	5 (6.0)	0.233

*mCRPC* metastatic castration resistant prostate cancer, *SD* standard deviation.

*p values are for comparisons between patients treated with radium-223 pre- vs. post-chemotherapy.

### Treatment patterns

In the overall cohort of 220 patients, 64 patients were treated with radium-223 pre-chemotherapy, 83 treated with radium-223 post-chemotherapy, and 73 were treated with radium-223 and no chemotherapy for mCRPC. Prior to the development of castration resistance, 28 (12.7%) patients received docetaxel, two (0.9%) received abiraterone acetate, and two (0.9%) received enzalutamide for hormone sensitive disease. Of the patients receiving radium-223 pre-chemotherapy and post-chemotherapy, 14 (21.9%) vs. 3 (3.6%) had received docetaxel for hormone sensitive disease, respectively. Of these 17 patients, 9 (52.9%) had metastases at the time of docetaxel administration. Among patients treated with radium-223 but no chemotherapy for mCRPC, 11 (15.1%) patients received docetaxel for hormone sensitive disease, and 9 (81.2%) had metastases at that time. (Supplementary Table 1).

Overall, 122 (55.5%) patients received bone-health agents at initiation of first-line mCRPC therapy, including zoledronic acid (*n* = 69), denosumab (*n* = 50), pamidronate (*n* = 2) and alendronate (*n* = 2); a similar number received bone-health agents at initiation of radium-223. Among the 64 patients treated with radium-223 pre-chemotherapy, 34 (53.1%) received some bone-health agent at initiation of first-line mCRPC therapy and 40 (62.5%) at initiation of radium-223. Among the 83 patients treated with radium-223 post-chemotherapy, this was 43 (51.8%) and 41 (49.4%), respectively.

For radium-223, mean line of mCRPC therapy was 3rd and 5th when given pre- and post-chemotherapy, respectively (*p* < 0.001) (Table 2). Mean number of radium-223 injections was 5.4 for the overall patient population, and 5.3 vs. 4.3 for patients treated with radium-223 pre- vs. post-chemotherapy for mCRPC (*p* < 0.001). For patients treated with radium-223 pre- vs. post-chemotherapy, a significantly higher proportion completed all 6 radium-223 injections (76.6% vs. 46.3%, *p* < 0.001) and a lower proportion discontinued due to disease progression (21.9% vs. 37.8%, *p* = 0.039) or adverse events (3.1% vs. 14.6%, *p* = 0.019). For patients treated with radium-223 and no chemotherapy, mean line of mCRPC therapy for radium-223 was 3rd and mean number of radium-223 injections was 4.3.

**Table 2:** Characteristics of radium-223 and chemotherapy treatment for mCRPC.

	Patients treated with radium-223 (*N* = 220)	Patients treated with radium-223 and no chemotherapy (*N* = 73)	Patients treated with radium-223 pre-chemotherapy (*N* = 64)	Patients treated with radium-223 post-chemotherapy (*N* = 83)	*p* value^a^
Radium-223					
Line number, mean ± SD [median]	3.4 ± 1.6 [3.0]	2.5 ± 0.8 [2.0]	2.8 ± 0.8 [3.0]	4.7 ± 1.7 [4.0]	<0.001*
Line number, *n* (%)					
1	2 (3.8)	6 (8.2)	4 (6.3)	0 (0.0)	0.034*
2	12 (22.6)	34 (46.6)	15 (23.4)	4 (4.8)	<0.001*
3	29 (54.7)	27 (37.0)	34 (53.1)	11 (13.3)	<0.001*
4	9 (17.0)	6 (8.2)	10 (15.6)	30 (36.1)	0.006*
5	1 (1.9)	0 (0.0)	1 (1.6)	20 (24.1)	<0.001*
6	0 (0.0)	0 (0.0)	0 (0.0)	10 (12.0)	0.005*
≥7	0 (0.0)	0 (0.0)	0 (0.0)	8 (9.6)	0.010*
Treatment setting, *n* (%)					
Clinical trial	1 (1.9)	0 (0.0)	1 (1.6)	2 (2.4)	>0.999
EAP	0 (0.0)	0 (0.0)	0 (0.0)	0 (0.0)	>0.999
Neither	52 (98.1)	73 (100.0)	63 (98.4)	80 (96.4)	0.633
Unknown	0 (0.0)	0 (0.0)	0 (0.0)	1 (1.2)	>0.999
Number of injections, mean ± SD [median]	5.4 ± 1.3 [6.0]	4.3 ± 1.8 [5.0]	5.3 ± 1.4 [6.0]	4.3 ± 1.9 [5.0]	<0.001*
Number of patients with dose delay, *n* (%)	52 (98.1)	69 (94.5)	62 (96.9)	79 (95.2)	0.697
Number of patients with treatment discontinuation, *n* (%)	53 (100.0)	72 (98.6)	64 (100.0)	82 (98.8)	>0.999
Reason for treatment discontinuation, *n* (%)^b^					
Course of therapy complete	42 (79.2)	33 (45.8)	49 (76.6)	38 (46.3)	<0.001*
Disease progression	11 (20.8)	15 (20.8)	14 (21.9)	31 (37.8)	0.039*
Adverse events	1 (1.9)	13 (18.1)	2 (3.1)	12 (14.6)	0.019*
Decreased quality of life	0 (0.0)	6 (8.3)	0 (0.0)	6 (7.3)	0.035*
Insurance reasons	0 (0.0)	1 (1.4)	0 (0.0)	0 (0.0)	>0.999
Drug interactions	0 (0.0)	0 (0.0)	0 (0.0)	0 (0.0)	>0.999
Patient refusal/decision	0 (0.0)	5 (6.9)	0 (0.0)	3 (3.7)	0.256
Other medical reasons	0 (0.0)	5 (6.9)	0 (0.0)	1 (1.2)	>0.999
Other	1 (1.9)	8 (11.1)	1 (1.6)	7 (8.5)	0.079
Unknown	0 (0.0)	2 (2.8)	0 (0.0)	2 (2.4)	0.504
First chemotherapy	n/a	n/a			
Type of chemotherapy, *n* (%)					
IV chemotherapy			64 (100.0)	83 (100.0)	>0.999
Docetaxel			49 (76.6)	77 (92.8)	0.005*
Number of cycles, mean ± SD [median]			5.3 ± 3.2 [5.0]	6.9 ± 4.3 [6.0]	0.063
Cabazitaxel			12 (18.8)	5 (6.0)	0.017*
Carboplatin			1 (1.6)	0 (0.0)	0.435
Mitoxantrone			0 (0.0)	0 (0.0)	>0.999
Cyclophosphamide			0 (0.0)	0 (0.0)	>0.999
Estramustine			0 (0.0)	0 (0.0)	>0.999
Other^c^			4 (6.3)	1 (1.2)	0.167
Oral chemotherapy			0 (0.0)	1 (1.2)	>0.999
Estramustine			0 (0.0)	1 (1.2)	>0.999
Cyclophosphamide			0 (0.0)	0 (0.0)	>0.999
Other^d^			0 (0.0)	1 (1.2)	>0.999
Number of chemotherapy administrations, mean ± SD [median]			9.0 ± 6.6 [8.0]	9.2 ± 6.4 [8.0]	0.768
Line number, mean ± SD [median]			4.2 ± 0.9 [4.0]	2.1 ± 1.0 [2.0]	<0.001*
Line number, *n* (%)					
1			0 (0.0)	30 (36.1)	<0.001*
2			2 (3.1)	22 (26.5)	0.098
3			11 (17.2)	24 (28.9)	<0.001*
4			29 (45.3)	6 (7.2)	<0.001*
5			18 (28.1)	1 (1.2)	0.080
6			3 (4.7)	0 (0.0)	>0.999
≥7			0 (0.0)	0 (0.0)	>0.999
Number of patients with treatment modification not including discontinuation, *n* (%)			0 (0.0)	3 (3.6)	0.258
Number of patients with treatment discontinuation, *n* (%)			62 (96.9)	83 (100.0)	0.188
Reason for treatment discontinuation, *n* (%)^b^					
Disease progression			41 (66.1)	57 (68.7)	0.746
Adverse events			19 (30.6)	21 (25.3)	0.476
Course of therapy complete			14 (22.6)	20 (24.1)	0.831
Insurance reasons			6 (9.7)	6 (7.2)	0.597
Decreased quality of life			5 (8.1)	3 (3.6)	0.288
Patient refusal/decision			1 (1.6)	1 (1.2)	>0.999
Drug interactions			0 (0.0)	0 (0.0)	>0.999
Other medical reasons			0 (0.0)	0 (0.0)	>0.999
Other			0 (0.0)	3 (3.6)	0.261
Unknown			0 (0.0)	1 (1.2)	>0.999

*EAP* expanded access program, *IV* intravenous, *mCRPC* metastatic castration resistant prostate cancer, *n/a* not applicable, *SD* standard deviation.

*indicates statistical significance (*p*-value < 0.05).

^a^*p* values are for comparisons between patients treated with radium-223 pre- vs. post-chemotherapy.

^b^Calculated from the number of patients who discontinued.

^c^Includes cisplatin, docetaxel+carboplatin, and etoposide.

^d^Includes exisulind.

For patients treated with radium-223 and chemotherapy for mCRPC, docetaxel was the most common first chemotherapy treatment (76.6% vs. 92.8% for radium-223 pre- vs. post-chemotherapy, *p* = 0.005) (Table 2). On average, chemotherapy was given as 4th and 2nd line mCRPC therapy in patients treated with radium-223 pre- and post-chemotherapy, *p* < 0.001. Median number of cycles of chemotherapy was 8 for both patients treated with radium-223 pre- and post-chemotherapy.

Ninety-two (41.8%) patients were treated with radium-223 in combination with another mCRPC therapy (Table 3). On average, radium-223 was given as 4th line mCRPC therapy for both radium-223 combination therapy and monotherapy (*p* = 0.180). The mean number of radium-223 injections was 5.3 vs. 4.3 for patients treated with radium-223 combination therapy vs. radium-223 monotherapy (*p* = 0.003). Most commonly radium-223 was given in combination with abiraterone acetate (*n* = 40, 43.5%) or enzalutamide (*n* = 48, 52.2%). Treatment overlapped with the entire duration of radium-223 in the majority of patients (*n* = 63, 68.5%); 19 (20.7%) patients started another agent after radium-223 initiation, while another 19 (20.7%) patients initiated radium-223 while on an established therapy.

**Table 3:** Radium-223 treatment for patients treated with radium-223 combination vs. monotherapy.

	Patients treated with radium-223 combination therapy (*N* = 92)	Patients treated with radium-223 monotherapy (*N* = 128)	*p* value
Radium-223			
Line number, mean ± SD [median]	3.8 ± 1.7 [3.0]	4.0 ± 1.6 [4.0]	0.180
Line number, *n* (%)			
1	1 (1.5)	3 (3.7)	0.630
2	9 (13.8)	10 (12.2)	0.767
3	25 (38.5)	20 (24.4)	0.066
4	17 (26.2)	23 (28.0)	0.798
5	6 (9.2)	15 (18.3)	0.119
6	3 (4.6)	7 (8.5)	0.513
≥7	4 (6.2)	4 (4.9)	0.733
Treatment setting, *n* (%)			
Clinical trial	3 (4.6)	0 (0.0)	0.084
EAP	0 (0.0)	0 (0.0)	>0.999
Neither	61 (93.8)	82 (100.0)	0.036*
Unknown	1 (1.5)	0 (0.0)	0.442
Number of injections, mean ± SD [median]	5.3 ± 1.3 [6.0]	4.3 ± 1.9 [6.0]	0.003*
Number of patients with dose delay, n (%)	63 (96.9)	78 (95.1)	0.694
Number of patients with treatment discontinuation, *n* (%)	64 (98.5)	82 (100.0)	0.442
Reason for treatment discontinuation, *n* (%)^a^			
Course of therapy complete	45 (70.3)	42 (51.2)	0.020*
Disease progression	19 (29.7)	26 (31.7)	0.793
Adverse events	2 (3.1)	12 (14.6)	0.019*
Decreased quality of life	1 (1.6)	5 (6.1)	0.231
Insurance reasons	0 (0.0)	0 (0.0)	>0.999
Drug interactions	0 (0.0)	0 (0.0)	>0.999
Patient refusal/decision	1 (1.6)	2 (2.4)	>0.999
Other medical reasons	1 (1.6)	0 (0.0)	0.438
Other	2 (3.1)	6 (7.3)	0.466
Unknown	0 (0)	2 (2.4)	0.504
Combination Radium-223 Treatment for mCRPC			
Overlapping treatment, *n* (%)			
Abiraterone acetate	40 (43.5)	–	
Enzalutamide	48 (52.2)	–	
IV Chemotherapy	13 (14.1)	–	
Cabazitaxel	2 (2.2)	–	
Docetaxel	9 (9.8)	–	
Other	2 (2.2)	–	
Other	7 (7.6)		
Investigational	4 (4.3)		
Pembrolizumab	3 (3.3)		
Overlap type, *n*(%)			
Duration of radium-223 treatment	63 (68.5)	–	
Abiraterone acetate	21 (22.8)	–	
Enzalutamide	30 (32.6)	–	
Starting and ending within radium-223 treatment	5 (5.4)	–	
Abiraterone acetate	3 (3.3)	–	
Enzalutamide	2 (2.2)	–	
Starting within radium-223 treatment	19 (20.7)	–	
Abiraterone acetate	4 (4.3)	–	
Enzalutamide	11 (12.0)	–	
Ending within radium-223 treatment	19 (20.7)	–	
Abiraterone acetate	12 (13.0)	–	
Enzalutamide	5 (5.4)	–	

*EAP* expanded access program, *IV* intravenous, *mCRPC* metastatic castration resistant prostate cancer, *SD* standard deviation.

*indicates statistical significance (*p*-value < 0.05).

^a^Calculated from the number of patients who discontinued.

### Survival & SSEs

In the total population, median OS measured from first-line mCRPC treatment initiation and the initiation of radium-223 was 33.0 months (95% CI 28.8, 36.5) and 12.4 months (95% CI 10.2, 13.9), respectively (Table 4). There was no significant difference in OS from initiation of mCRPC treatment between patients who were treated with radium-223 pre- vs. post-chemotherapy (39.4 months [95% CI 33.0, 48.8] vs. 37.4 months [95% CI 32.0, 43.5]), nor when patients treated with abiraterone acetate or enzalutamide combined with radium-223 were excluded (42.6 months [95% CI 33.0, 55.1] vs. 37.5 months [95% CI 32.4, 44.7]). There were no significant differences in OS from mCRPC treatment initiation between patients treated with radium-223 in combination therapy vs. monotherapy (35.2 months [95% CI 27.9, 43.4] vs. 32.0 months [95% CI 26.9, 36.0]). OS for patients treated with radium-223 either pre- or post-chemotherapy was prolonged among patients who completed 5–6 vs. 1–4 injections of radium-223 (median OS was 18.7 months [95% CI 16.2, 22.8] vs. 5.9 months [95% CI 4.7, 7.3] from radium-223 initiation, and 47.5 months [95% CI 41.9, 55.0] vs. 27.3 months [95% CI 20.4, 32.4] from first-line mCRPC treatment).

**Table 4:** Overall survival for mCRPC patients treated with radium-223.

	*N*	Median OS months [95% CI]
OS measured from initiation of radium-223		
All patients treated with radium-223	220	12.4 [10.2, 13.9]
OS measured from initiation of first-line mCRPC treatment		
All patients treated with radium-223	220	33.0 [28.8, 36.5]
Patients treated with radium-223 pre- or post-chemotherapy	147	38.7 [34.4, 44.2]
Patients treated with radium-223 pre-chemotherapy	64	39.4 [33.0, 48.8]
Excluding patients treated with radium-223 in combination with abiraterone acetate or enzalutamide	30	42.6 [33.0, 55.1]
Patients treated with radium-223 post-chemotherapy	83	37.4 [32.0, 43.5]
Excluding patients treated with radium-223 in combination with abiraterone acetate or enzalutamide	64	37.5 [32.4, 44.7]
Patients treated with radium-223 combination therapy	92	35.2 [27.9, 43.3]
Abiraterone acetate	40	29.0 [23.2, 43.3]
Enzalutamide	48	33.1 [27.2, 43.3]
IV chemotherapy	13	49.6 [36.2, 69.0]
Other	5	48.4 [18.8, NR]
Patients treated with radium-223 monotherapy	128	32.0 [26.9, 36.0]

*CI* confidence interval, *IV* intravenous, *mCRPC* metastatic castration resistant prostate cancer, *NR* not reached, *OS* overall survival.

Overall, 32 (14.5%) patients experienced an SSE while on first-line mCRPC therapy, and 29 (13.2%) while on radium-223 treatment. For patients treated with radium-223 pre- vs. post-chemotherapy, 11 (17.2%) vs. 7 (8.4%) of patients experienced an SSE while on first-line mCRPC therapy, and 7 (10.9%) vs. 10 (12.0%) while on radium-223 treatment.

## Discussion

This retrospective analysis of patients treated with radium-223 highlights that mCRPC data on optimal mCRPC treatment sequencing is limited, and variations in treatment sequencing patterns exist. We demonstrate that while radium-223 completion rates differ in those receiving treatment pre- and post- chemotherapy, OS was similar in both groups. As treatment options populate the hormone sensitive space for patients with advanced disease, the sequencing of therapies for CRPC becomes more complex and treatment options limited.

The phase 3 ALSYMPCA trial which led to FDA approval of radium-223 was designed to study patients who were previously treated with chemotherapy or declined chemotherapy, and 57% of patients were previously treated with docetaxel [8]. The current study describes a wide range of treatment patterns and their outcomes regardless of treatments that preceded or followed radium-223. In our study, 29% of patients received radium-223 pre-chemotherapy, and 93% received abiraterone acetate or enzalutamide prior to radium-223, providing information not captured in the ALSYMPCA trial, which was conducted prior to the approval of those treatments.

OS results from the current study are in line with what has been observed previously. In the ALSYMPCA trial of radium-223 vs. placebo, median OS was 14.9 vs. 11.3 months [9]. A phase II prospective EAP followed ALSYMPCA to monitor radium-223 safety, and allowed for pre-treatment with abiraterone acetate, enzalutamide, cabazitaxel and docetaxel and concurrent treatment with abiraterone acetate and enzalutamide, differing from the patient population in ALSYMPCA. Median OS was 17 months [15]. Information on OS from patients with variable treatment patterns is important to complement data from clinical trials. Data reported in this study come from a wider range of patients who are eligible for trials, which often underrepresent elderly patients and those with numerous comorbidities [16].

mCRPC treatment sequencing will evolve as therapies are also approved for hormone sensitive and nonmetastatic CRPC. The data in the current study include patients who were diagnosed with mCRPC from 2003–2017, when transitions in the hormone sensitive landscape were underway [16]. Docetaxel added to androgen deprivation therapy (ADT) for metastatic hormone sensitive disease became standard in 2015 [17–19], the abiraterone acetate label expanded for treatment of metastatic high-risk hormone sensitive prostate cancer in 2018 [20], approval for both apalutamide and enzalutamide expanded to include metastatic hormone sensitive disease in 2019 [21–23], and darolutamide was approved for nonmetastatic CRPC in 2019 [24]. Radium-223 in mCRPC treatment sequencing therefore may shift due to approval of other therapies in earlier stages. For example, in the European Union, radium-223 is now indicated after two lines of systemic therapy for mCRPC [25]. Given that visceral metastases can develop as CRPC disease progression occurs, there is rationale for use of radium-223 early in the treatment course prior to development of visceral metastases [1].

Sequencing of radium-223 is being studied in the global REASSURE study [13]. An interim analysis showed potential benefits of earlier radium-223; patients who had not previously received chemotherapy were more likely to complete 6 radium-223 injections than those who had [13], consistent with this study. Furthermore, patients treated with radium-223 without prior chemotherapy tended to have better safety profiles and less treatment discontinuation [13, 25]. An EAP showed completion of 5–6 vs. 1–4 injections of radium-223 was associated with prolonged survival (median OS was not estimable vs. 7.5 months for 5–6 vs. 1–4 injections) [15], similar to results found in this study. Importantly, the current study shows that patients who were treated with radium-223 pre- and post-chemotherapy were each treated with a median of 8 cycles of chemotherapy, indicating that radium-223 does not compromise future chemotherapy treatment.

Combination therapy vs. monotherapy was not associated with a survival advantage in this study; however, there were many variations in combinations, and the numbers are small, so differences may not have been detected. Duration of the combination also varied (e.g., overlapping for all of radium-223 treatment or only a portion). It is also noted that starting/stopping combination therapy was at the treating physician’s discretion, thus results presented here are descriptive.

There were 40 patients in our study who were treated with radium-223 combined with abiraterone acetate. These data preceded the recommendation against the combination following the ERA 223 trial which showed that the combination did not improve SSE-free survival and was associated with increased bone fractures [26], although a post hoc analysis found that use of bone-health agents at baseline was far less common among patients who experienced fractures [27]. ERA 223 followed the phase II trial eRADicAte which demonstrated that patients treated with 6 cycles of radium-223 with abiraterone acetate experienced improved quality of life and pain and no unexpected toxicities [28]. An ongoing study of enzalutamide with radium-223 (NCT03344211) [29] will yield additional results on radium-223 treatment combined with next generation ADT.

Ongoing trials are assessing radium-223 combined with other treatments, including docetaxel for mCRPC in the DORA trial [30], sipuleucel-T for asymptomatic or minimally symptomatic bone-mCRPC in a phase III study [31], stereoactive ablative radiation for oligometastatic prostate cancer in the RAVENS phase II study [32], and pembrolizumab for mCRPC in a phase II study [33]. Radium-223 combination with olaparib is also being examined (COMRADE trial) [34]. PARP-inhibitors such as olaparib may be particularly beneficial to men with mutations in DNA repair genes [16] as demonstrated by the phase III PROfound study, among men with mCRPC with alterations in homologous recombination repair genes [35]. Cancer cells with defects in recombination DNA repair genes are especially susceptible to PARP inhibitors because these agents inhibit PARP repair of single strand DNA breaks [36]. While radium-223 induces double strand DNA breaks, single strand DNA breaks also occur, suggesting combination with PARP inhibition may be a rational treatment strategy; case reports suggest high frequency of DNA repair pathway alterations in patients who responded to radium-223 (4 of 10), with prospective studies planned to confirm this [37, 38]. PARP-inhibitors olaparib, rucaparib, and niraparib were granted breakthrough therapy designation in 2018–2019 for mCRPC with certain mutations [39–41]. Future treatments for mCRPC may become more biomarker-driven.

Bone-health agents were also assessed in the current study. A post hoc analysis of the ALSYMPCA trial showed there was a greater benefit of radium-223 on the time to first SSE among patients who received bisphosphonates at baseline [42], and there is emerging evidence that the addition of these agents may confer further clinical benefit [43]. In this study, only slightly more than half of patients received bone-health agents, consistent with an observational study in the US that reported 61%, underlining their underutilization [44]. While bone-health agents are generally well tolerated there is risk of hypocalcemia at initiation and low risk of osteonecrosis of the jaw [43].

There are limitations to the current study. Data were collected retrospectively and limited to medical charts. OS analyses did not adjust for the use of other mCRPC treatments, as this study aimed to be descriptive in nature and hypothesis-generating. The results reported in this study are reflective of practice patterns at a single institution and may not be generalizable. This retrospective study of treatment of mCRPC with radium-223 provides insight into the management and clinical outcomes of patients treated in routine clinical practice and demonstrates that radium-223 treatment prior to chemotherapy may be associated with higher completion rate of radium-223 without compromising subsequent chemotherapy. Additional prospective studies that evaluate the optimal treatment sequence are warranted to improve outcomes for patients. A better understanding of tumor biology and predictive biomarkers will be critical for treatment selection for patients with advanced disease.

## Supplementary information

Supplemental table
