# Supplementary material for: Treatment of metastatic castration resistant prostate cancer with radium-223: a retrospective study at a US tertiary oncology center
Source: Prostate Cancer Prostatic Dis. 2020 Aug 19;24(1):210–9. doi: 10.1038/s41391-020-00271-7 (PMC8012208; doi:10.1038/s41391-020-00271-7)
Supplement: Supplementary file 1 — Supplemental table [file 41391_2020_271_MOESM1_ESM.docx]

Treatments that patients received prior to mCRPC, including radical prostatectomy, and treatments in the hormone sensitive phase and in the CRPC phase, are shown below.

| **Supplementary Table 1.** Treatments Prior to mCRPC | | | | | | |
| --- | --- | --- | --- | --- | --- | --- |
|  | Patients treated with radium-223 (N=220) | Patients treated with radium-223 and no chemotherapy (N=73) | Patients treated with radium-223 pre-chemotherapy (N=64) | Patients treated with radium-223 post-chemotherapy (N=83) | p-value^1^ |  |
| Radical prostatectomy, n (%) | 68 (30.9) | 12 (16.4) | 25 (39.1) | 31 (37.3) | 0.832 |  |
| Time to radical prostatectomy from PC diagnosis (month), mean ± SD [median] | 3.1 ± 2.6 [2.4] | 2.4 ± 1.6 [2.1] | 3.4 ± 3.2 [2.5] | 3.2 ± 2.6 [2.4] | 0.946 |  |
|  |  |  |  |  |  |  |
| *Treatments received in hormone sensitive phase* |  |  |  |  |  |  |
| First generation anti-androgens or androgen suppressing drugs, n (%) |  |  |  |  |  |  |
| Bicalutamide | 176 (80.0) | 54 (74.0) | 57 (89.1) | 65 (78.3) | 0.085 |  |
| Flutamide | 5 (2.3) | 2 (2.7) | 1 (1.6) | 2 (2.4) | >0.999 |  |
| Nilutamide | 3 (1.4) | 0 (0.0) | 1 (1.6) | 1 (1.2) | >0.999 |  |
| Ketoconazole | 2 (0.9) | 1 (1.4) | 1 (1.6) | 1 (1.2) | >0.999 |  |
| Estrogen | 1 (0.5) | 0 (0.0) | 1 (1.6) | 0 (0.0) | 0.435 |  |
| Other | 0 (0.0) | 0 (0.0) | 0 (0.0) | 0 (0.0) | >0.999 |  |
| None | 39 (17.7) | 19 (26.0) | 7 (10.9) | 13 (15.7) | 0.407 |  |
| Unknown | 2 (0.9) | 0 (0.0) | 0 (0.0) | 2 (2.4) | 0.505 |  |
| Abiraterone acetate, n (%) | 2 (0.9) | 0 (0.0) | 0 (0.0) | 2 (2.4) | 0.505 |  |
| Enzalutamide, n (%) | 2 (0.9) | 1 (1.4) | 0 (0.0) | 1 (1.2) | >0.999 |  |
| Docetaxel, n (%) | 28 (12.7) | 11 (15.1) | 14 (21.9) | 3 (3.6) | <0.001* |  |
| Patients with metastatic disease, n (%) | 18 (64.3) | 9 (81.8) | 9 (64.3) | 0 (0.0) | 0.082 |  |
| Number of cycles given, including initial administration, mean ± SD [median] | 5.1 ± 2.8 [6.0] | 5.7 ± 0.6 [6.0] | 4.4 ± 3.9 [6.0] | 6.0 ± 0.0 [6.0] | 0.144 |  |
| Other chemotherapy, n (%) |  |  |  |  |  |  |
| Cabazitaxel | 0 (0.0) | 0 (0.0) | 0 (0.0) | 0 (0.0) | >0.999 |  |
| Mitoxantrone | 0 (0.0) | 0 (0.0) | 0 (0.0) | 0 (0.0) | >0.999 |  |
| Cyclophosphamide | 0 (0.0) | 0 (0.0) | 0 (0.0) | 0 (0.0) | >0.999 |  |
| Estramustine | 2 (0.9) | 2 (2.7) | 0 (0.0) | 0 (0.0) | >0.999 |  |
| Carboplatin | 1 (0.5) | 1 (1.4) | 0 (0.0) | 0 (0.0) | >0.999 |  |
| Other^2^ | 2 (0.9) | 1 (1.4) | 0 (0.0) | 1 (1.2) | >0.999 |  |
| Apalutamide, n (%) | 0 (0.0) | 0 (0.0) | 0 (0.0) | 0 (0.0) | >0.999 |  |
| Pembrolizumab, n (%) | 0 (0.0) | 0 (0.0) | 0 (0.0) | 0 (0.0) | >0.999 |  |
| Investigational therapies^3^, n (%) | 6 (2.7) | 1 (1.4) | 1 (1.6) | 4 (4.8) | 0.388 |  |
|  |  |  |  |  |  |  |
| *Treatments received in CRPC phase* |  |  |  |  |  |  |
| First generation anti-androgens or androgen suppressing drugs, n (%) |  |  |  |  |  |  |
| Bicalutamide | 41 (18.9) | 9 (12.7) | 13 (20.3) | 19 (23.2) | 0.679 |  |
| Nilutamide | 20 (9.2) | 5 (7.0) | 4 (6.3) | 11 (13.4) | 0.157 |  |
| Ketoconazole | 8 (3.7) | 2 (2.8) | 1 (1.6) | 5 (6.1) | 0.231 |  |
| Flutamide | 2 (0.9) | 1 (1.4) | 0 (0.0) | 1 (1.2) | >0.999 |  |
| Estrogen | 1 (0.5) | 0 (0.0) | 0 (0.0) | 1 (1.2) | >0.999 |  |
| Other^4^ | 2 (0.9) | 1 (1.4) | 0 (0.0) | 1 (1.2) | >0.999 |  |
| None | 156 (71.9) | 55 (77.5) | 49 (76.6) | 52 (63.4) | 0.088 |  |
| Unknown | 11 (5.0) | 4 (5.5) | 1 (1.6) | 6 (7.2) | 0.138 |  |
| Abiraterone acetate, n (%) | 10 (4.6) | 2 (2.8) | 3 (4.7) | 5 (6.1) | >0.999 |  |
| Enzalutamide, n (%) | 4 (1.8) | 1 (1.4) | 2 (3.1) | 1 (1.2) | 0.582 |  |
| Docetaxel, n (%) | 3 (1.4) | 1 (1.4) | 1 (1.6) | 1 (1.2) | >0.999 |  |
| Number of cycles given, including initial administration, mean ± SD [median] | 5.7 ± 2.1 [5.0] | 4.0 ± n/a [4.0] | 8.0 ± [8.0] | 5.0 ± [5.0] | >0.999 |  |
| Other chemotherapy, n (%) |  |  |  |  |  |  |
| Cabazitaxel | 1 (0.5) | 0 (0.0) | 1 (1.6) | 0 (0.0) | 0.438 |  |
| Mitoxantrone | 1 (0.5) | 0 (0.0) | 1 (1.6) | 0 (0.0) | 0.438 |  |
| Cyclophosphamide | 0 (0.0) | 0 (0.0) | 0 (0.0) | 0 (0.0) | >0.999 |  |
| Estramustine | 0 (0.0) | 0 (0.0) | 0 (0.0) | 0 (0.0) | >0.999 |  |
| Carboplatin | 0 (0.0) | 0 (0.0) | 0 (0.0) | 0 (0.0) | >0.999 |  |
| Other | 0 (0.0) | 0 (0.0) | 0 (0.0) | 0 (0.0) | >0.999 |  |
| Apalutamide, n (%) | 0 (0.0) | 0 (0.0) | 0 (0.0) | 0 (0.0) | >0.999 |  |
| Pembrolizumab, n (%) | 0 (0.0) | 0 (0.0) | 0 (0.0) | 0 (0.0) | >0.999 |  |
| Investigational therapies^5^, n (%) | 5 (2.3) | 0 (0.0) | 1 (1.6) | 4 (4.8) | 0.388 |  |
|  |  |  |  |  |  |  |
| Abbreviations: mCRPC, metastatic castration resistant prostate cancer; n/a, not applicable; SD, standard deviation  ^1^p-values are for comparisons between patients treated with radium-223 pre- vs. post-chemotherapy.  ^2^Includes docetaxel + carboplatin.  ^3^Includes everolimus, rosiglitazone, celecoxib, ATN224, and imatinib.  ^4^Includes conjugated estrogens.  ^5^Includes everolimus, sipuleucel-T, and OGX427. | | | | | | |
